# The alternative complement pathway revisited

**DOI:** 10.1111/j.1582-4934.2008.00350.x

**Published:** 2008-04-15

**Authors:** Morten Harboe, Tom Eirik Mollnes

**Affiliations:** Institute of Immunology, University of Oslo and Rikshospitalet University HospitalOslo, Norway

**Keywords:** complement activation, classical pathway, lectin pathway, alternative pathway, amplification

## Abstract

Alternative pathway amplification plays a major role for the final effect of initial specific activation of the classical and lectin complement pathways, but the quantitative role of the amplification is insufficiently investigated. In experimental models of human diseases in which a direct activation of alternative pathway has been assumed, this interpretation needs revision placing a greater role on alternative amplification. We recently documented that the alternative amplification contributed to 80–90% of C5 activation when the initial activation was highly specific for the classical pathway. The recent identification of properdin as a recognition factor directly initiating alternative pathway activation, like C1q in the classical and mannose-binding lectin in the lectin pathway, initiates a renewed interest in the reaction mechanisms of complement. Complement and Toll-like receptors, including the CD14 molecule, are two main upstream recognition systems of innate immunity, contributing to the inflammatory reaction in a number of conditions including ischaemia-reperfusion injury and sepsis. These systems act as ‘double-edged swords’, being protective against microbial invasion, but harmful to the host when activated improperly or uncontrolled. Combined inhibition of complement and Toll-like receptors/CD14 should be explored as a treatment regimen to reduce the overwhelming damaging inflammatory response during sepsis. The alternative pathway should be particularly considered in this regard, due to its uncontrolled amplification in sepsis. The alternative pathway should be regarded as a dual system, namely a recognition pathway principally similar to the classical and lectin pathways, and an amplification mechanism, well known, but quantitatively probably more important than generally recognized.

- Introduction
- Methods to study and modify the alternative pathway
  - Inhibition by monoclonal antibodies
  - Knockout models
- Influence of AP amplification on the effect of specific activation of the other initial pathways
  - Direct activation *versus* amplification
  - Mechanisms of amplification
- Properdin
  - Control protein in the AP amplification loop
  - Recognition molecule in AP
- Influence of AP in deficiency states
  - Factor H deficiency and variants
  - C2 bypass in CP and LP
  - Properdin deficiency
- Complement in sepsis
- Concluding remarks

## Introduction

**Fig. 1:**
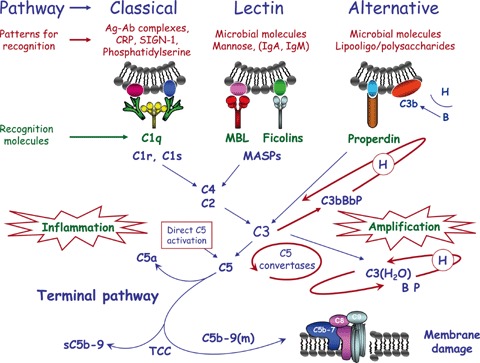
Complement activation proceeds through three distinct initial pathways. Patterns for recognition are indicated in red and recognition molecules in green. Properdin has a dual role in the alternative pathway. Activation is induced on a foreign surface favouring binding of factor B instead of factor H as indicated in the upper right. The proposed mechanism for reaction of properdin with a target on the foreign surface recruits fluid-phase C3b inducing assembly of AP C3 convertase [27] is indicated with a blue line pointing to C3. Amplification mechanisms are indicated in thick red lines: The slow spontaneous activation involving C3(H_2_O) reacting with factor B, ‘tick-over’, is controlled and inhibited by factor H. The second function of properdin is to stabilize the C3bBb complex promoting self-amplification in the other loop. These C3-directed reactions all promote C5 convertase amplification indicated with the third, circular loop. Direct C5 activation [16] is indicated in the red square. Formation of C5b initiates assembly of the components of the common terminal pathway with formation of the terminal complement complex TCC forming sC5b-9 in the fluid phase or C5b-9(m), when incorporated into a nearby membrane, leading to sub-lytic attack or lysis.

Complement activation proceeds through three distinct pathways as illustrated in Fig. 1. The classical pathway (CP) and the lectin pathway (LP) generate a common C3 convertase, C4b2a, but are initially activated by distinct recognition mechanisms.

CP is mainly activated by C1q reacting with antibodies bound to antigen [1, 2] and also by other agents, such as C-reactive protein (CRP) [3, 4], SIGN-R1, a lectin that captures microbial poly-saccharides in the spleen [5], and phosphatidylserine being important for efficient removal of apoptotic cells [6].

LP is mainly activated by mannose-binding lectin (MBL) interacting with carbohydrate structures on microbial surfaces and by ficolins with different fine carbohydrate binding specificity [7–11]. MBL reacts in particular with terminal mannose residues but also with other sugars, like N-acetyl-D-glucosamine, while ficolin 2 and 3 are pattern recognition molecules for acetyl groups.

The alternative pathway (AP) is slowly activated spontaneously by hydrolysis of the internal C3 thioester bond [12–14] and further triggered by contact with various proteins, lipids and carbohydrate structures on microorganisms and other foreign surfaces [1, 15].

Activation of the initial pathways results in formation of C5 convertases splitting C5. C5a is a potent anaphylatoxin. C5b induces assembly of the later components into the terminal C5b-9 complement complex, TCC [1, 2]. The terminal pathway is biologically highly potent and responsible for much of the inflammatory reaction induced by initial activation, particularly through the C5a receptor. A reduced AP amplification will thus in most cases substantially attenuate the effects of terminal pathway activation, although a direct C5 activation pathway without prior C3 involvement was recently postulated [16].

The complement system with a central position in acquired as well as innate immunity mediates a variety of effector functions. During the past decades, it has become evident that it is also an important mediator of tissue damage in disease [1, 2, 17], in particular in ischaemia/reperfusion (I/R) injury [18, 19]. Clinical evidence pointed to complement and immune complexes as critical players in mediating reperfusion injury [20]. Intestinal I/R injury could also be initiated by clonally specific natural IgM antibodies [21].

Results in early studies of C3 and C4 knockout (−/−) mice suggested an important role of CP or LP in inducing complement activation during skeletal muscle and intestinal I/R injury [22, 23]. However, in a later study performed with the same intestinal model applying factor D (fD) −/− mice, the AP was shown to be critically involved in the I/R injury [24]. We have suggested, however, that AP amplification might play a more critical role in CP-induced C5 activation than previously recognized. Thus, we designed a method highly specific for CP activation in order to quantify the role of the AP amplification. Using pathway-specific anti-fD inhibitory monoclonal antibody (mAb) 166-32 [25], more than 80% of C5a and TCC formation was inhibited after solid phase IgM- and solid- and fluid phase human aggregated IgG induced activation of CP [26]. A similar highly specific lectin pathway activation system was designed [15] and the AP amplification seems to play a decisive role regarding the final effect of the initial activation (Harboe and Mollnes, unpublished observations).

Recently, new data have appeared showing that properdin has two clearly distinct functions: in addition to enhance AP amplification by stabilization of the C3bBb complex, properdin is a recognition factor directly initiating AP activation [27–29]. Thus, AP should be regarded as a recognition pathway through properdin, like CP through C1q and LP through MBL and ficolins, in addition to being an amplification system for all three initial pathways (Fig. 1).

The main subject of this review is to consider this dichotomy of the AP and the major influence of AP amplification on the effect of complement activation in experimental models and human disease.

## Methods to study and modify the alternative pathway

### Inhibition by monoclonal antibodies

Factor D is a rate-limiting serine protease in the amplification loop of the alternative pathway [30] and a target of choice for effective, specific inhibition of AP. Fung *et al.* generated an anti-fD mAb (clone 166-32, IgG1) by immunizing mice with human fD and shown to inhibit AP activation completely [25]. The binding epitope of the antibody is located in the ‘methionine loop’ of fD (-CNRRTHHDGAITERLMC-) between Cys154 and Cys170 [26]. This epitope is distant from the catalytic site of the trypsin-like domain of fD in which there is extensive homology with other serine proteases of the complement system (C1r, C1s, MASP-1, MASP-2). The antibody-binding site may be responsible for the interaction between fD and factor B (fB). Extensive search revealed no significant homology of this epitope sequence with other known proteins. This is consistent with observations in haemolytic assays in which mAb 166–32 reacts specifically with fD and inhibits AP, but does not interfere with CP components.

Thurman *et al.* developed an inhibitory mAb (clone 1379, IgG1) to fB by immunization of fB −/− mice with a fusion protein comprised of the second and third short consensus repeat (SCR) domains of mouse fB linked to a mouse IgG1 Fc domain [31]. mAb 1379 reacts with an epitope on the surface of the SCR3 region of fB and prevents formation of the C3bBb complex leading to complete inhibition of AP *in vitro* and *in vivo*.

Gupta-Bansal *et al.* generated a total of 74 monoclonal anti-properdin antibodies by immunization of mice with human properdin and assay for ability to prevent properdin binding to C3b [32]. Eight of them were blocking antibodies efficiently inhibiting AP activation. These antibodies also reduced TCC formation after addition of protamine to heparinized blood suggesting that the AP amplification loop is a ‘major contributor to sC5b-9 formation after CP activation by heparin-protamine complexes in blood’[32].

### Knockout models

To generate fD knockout mice, Xu *et al.* constructed a gene-targeting vector inserting a *neo*^r^ (neomycin-resistance) gene cassette to disrupt exon 3 of the fD gene containing the Asp residue of the serine protease active centre [33]. In homozygous fD −/− mice, Northern blots of RNA showed the absence of fD message, and fD protein was not detectable in serum by Western blotting. Serum did not deposit C3 on zymosan particles in Mg/EGTA buffer and did not haemolyse rabbit erythrocytes, typical features of AP activation. Reconstitution with purified fD reversed these defects.

To generate fB knockout mice, Matsumoto *et al.* interrupted the murine fB gene by exchange of exon 3 through exon 7 with the *neo*^r^ gene [34]. This site was chosen to avoid compromising C2 expression; the 3′ end of the C2 gene overlaps fB promoter elements and is only about 100 bp upstream of the 5′ fB transcription start site. The deletion included exon 6 encoding the fD cleaving site required for fB activation. Disruption of the fB gene was confirmed by Northern blotting and studies of fB protein synthesis in cell culture. In serum from fB −/− mice, intact fB protein could not be detected and there was no AP activity. C3 was not deposited on zymosan in Mg/EGTA buffer, and purified mouse fB reversed the deficit. C3 and C4 protein and C2 mRNA levels were similar in wild-type and fB −/− mice confirming no effect on essential early CP components. The abstract states ‘Classical pathway-dependent total haemolytic activity was lower in fB-deficient than wild-type mice, possibly reflecting loss of the alternative pathway amplification loop’[34].

To generate properdin knockout mice, Kimura *et al.* targeted exon 3-5 of the properdin gene for disruption [28] because mutations in exon 4-6 of the human properdin gene are among those associated with type I properdin deficiency with absence of circulating properdin [35–37]. In properdin −/− mice, Northern blots showed absence of properdin mRNA and Western blots confirmed lack of properdin protein in plasma. *Salmonella* typhosa lipopolysaccharide (LPS) induced AP complement activation was abolished in serum from properdin −/− mice being restored with purified human properdin.

Stover *et al.* independently developed another properdin −/−mouse [29] showing similar properties; hemizygous male mice had no detectable properdin protein in serum and did not lyse rabbit red cells in the standard test for AP activity. Properdin in wild-type mouse serum was shown to associate directly with E. coli K12 DH5α exhibiting rough LPS while properdin −/− serum lacked this activity, again pointing to properdin as recognition unit in the AP.

## Influence of alternative pathway amplification on the effect of specific activation of the other initial pathways

Extensive studies in well-established murine models of intestinal I/R injury illustrate changing views on its pathogenesis, in particular regarding the role of the three complement pathways for induction of tissue damage.

Totally antibody-deficient Rag-1 knockout (−/−) mice did not develop I/R damage while reconstitution with pooled IgM isolated from wild-type sera restored injury [23], identifying IgM antibody as an essential inducer of tissue damage. Studies of C3 −/− and C4 −/− mice further supported an important role of CP or LP in inducing complement activation during skeletal muscle and intestinal I/R injury, but could not allocate the effect to one of these two pathways [22, 23].

In a separate study in the same model, fD −/− mice did not develop intestinal I/R injury [24], turning the attention to AP and its important role in development of local and remote tissue injury after gastrointestinal I/R. We have suggested, however, that absence of amplification *via* AP in fD −/− mice could reduce the complement-mediated inflammatory response and tissue injury, triggered by initial activation of the CP, but mainly effectuated by AP amplification and the potent terminal pathway. For this purpose, we designed a highly CP specific activation system and used pathway-specific anti-fD mAb to show that AP amplification plays a decisive role regarding the final effect of initial specific activation of CP. Indeed, selective blockade of AP by neutralization of fD with mAb 166-32 inhibited more than 80% of C5a and TCC formation induced by solid phase IgM and solid- and fluid-phase human aggregated IgG *via* CP [26]. To further investigate the role of AP amplification of initial complement activation, we designed a highly specific assay for activation of LP [15]. AP amplification seems to play a similar role regarding the final effect of the initial activation (Harboe and Mollnes, unpublished observations). Studies of C1q −/− and MBL −/− mice have been essential to identify the relative role of CP and LP in development of intestinal and myocardial I/R injury. MBL −/− mice were protected while C1q −/− mice were not, indicating a decisive role of LP in these conditions [38, 39].

The self-reactive clonal natural IgM antibody shown to initiate the I/R injury [21, 40] was recently demonstrated to react with a highly conserved region within non-muscle myosin heavy chain type II (NMHC-II) A and C [41]. When further experiments showed that murine MBL could bind to IgM, a novel model could be suggested in which intestinal I/R tissue injury is induced when LP is activated following recognition of ischaemic antigen by natural IgM antibody [42].

In skeletal muscle I/R CP and LP play different roles. Blockade of CP alone in C1q −/− mice protects against permeability oedema and remote pulmonary injury but not against histo-logic muscle injury while blocking of LP alone in MBL −/− mice protects against histological injury but not against permeability oedema or lung injury. Thus, activation of both pathways is probably needed to induce the full spectrum of injuries observed after skeletal muscle reperfusion injury [43]. Skeletal muscle I/R damage is specifically blocked by intravenous administration of amino acid peptides selected by a 12-mer phage display further identifying the pathogenic role of the natural clonal IgM antibody [44].

The relative importance of CP and LP in I/R injury may thus vary in different models. The basis for this variation remains to be shown, although initiation of the reaction by the clonal IgM antibody is a common feature. Variation in the content of the reactive epitope in different tissues may be an important feature to explain that apparently different mechanisms of complement activation occur in different organs. There are indications that the I/R injury in the heart is initiated by MBL and the LP [39], whereas renal I/R injury might be due to loss of regulators of the AP in the tubuli [45]. A common feature for any initial complement activation is AP amplification. Initial activation of both CP and LP generates a common C3 convertase, C4b2a, forming C3b, which is essential in starting up the amplification loop providing a rational explanation for the common AP amplification (Fig. 1).

Although the initial reactions in CP and LP are similar, there is a potential for four times more amplification *via* LP than CP in generation of C3/C5 convertases since it was demonstrated *in vitro* that LP activation deposited four times more C4 than CP activation [46]. Thus, production of proinflammatory products through LP might be a relatively more prominent contributor to inflammatory reactions if this also holds true for the physiological convertases formed *in vivo*.

### Direct activation *versus* amplification

To distinguish between two alternatives: *(i)* direct activation of AP and *(ii)* initial activation of CP or LP where the final effect to a large extent is determined by AP amplification is often difficult. In studies of specific LP activation of normal human serum by different amounts of mannan on the solid phase of ELISA plates [15], comparison between the effect of 3F8 inhibiting and 1C10 non-inhibiting anti-MBL mAbs [47] is very informative. Complete inhibition of TCC generation by 3F8 mAb indicates no direct effect on AP under these conditions of even high amounts of mannan on the solid phase (Harboe and Mollnes, unpublished observations). Comparison between the effect of selected inhibiting and non-inhibiting anti-C1q mAbs [48] provides similar information on the relative importance of CP and direct activation of AP by other compounds. In our view, a decisive importance of AP amplification is involved in several experimental models in which antibodies are involved in the initial activation. The MRL/lpr model of lupus nephritis [49, 50], arthritis in mice triggered by immune complex deposition [51], anti-phospholipid antibody-mediated foetal injury [31, 52, 53] and collagen antibody-induced arthritis in mice [54] are candidates for this interpretation.

By contrast, the striking complement activation during coronary bypass surgery has for a long time been a candidate for initial triggering of AP through contact between blood and the foreign surface with rapid formation of C3b. If factor H (fH) is bound, the surface will appear as ‘compatible’ while more exclusive binding of fB will initiate AP activation [55], consistent with the view of complement's ability to discriminate ‘self’ from ‘non-self’ illustrated in figure 2 in [1].

Conjugation of heparin to biomaterials improves biocompatibility. When polystyrene microtitre plates were incubated with human serum, C3 was deposited on the surface with activation of CP and AP. Heparin surface modification decreased complement activation related to an enhanced fH/factor I (fI)-dependent down-regulation of

C3b binding and decreased AP amplification [56]. Studies of complement activation in blood in contact with polystyrene by quartz crystal microbalance-dissipation analysis (QCM-D) showed that the surface was immediately covered with a plasma protein film of about 8 nm triggering limited CP activation followed by C3 binding and AP amplification [57]. Thus, initial triggering of CP, the effect of which is largely determined by AP amplification, also appears to be a central feature in these circumstances.

Experimental data indicate that anti-neutrophil cytoplasmic autoantibodies (ANCAs) induce glomerulonephritis and vasculitis through another mechanism. After injection of IgG anti-myeloper-oxidase (MPO-ANCA), C4 −/− mice developed disease comparable to wild-type mice while fB −/− mice developed no disease, indicating a primary role of AP in induction of the disease. Incubation of MPO-ANCA or anti-myeloperoxidase 3 ANCA with human neu-trophils stimulated the cells resulting in release of factors activating AP, citation: ‘thus initiating an inflammatory amplification loop that mediates the severe necrotizing inflammation of ANCA disease’[58].

### Mechanisms of amplification

Initial activation of CP and LP generates a common C3 convertase, C4b2a, splitting C3 into C3a and C3b. Upon formation of C3b the amplification loop of AP is induced on the activating surface and/or in the fluid phase. Different mechanisms of amplification in the fluid phase and on cell surfaces are discussed in more detail by Lutz and Jelezarova [59] and visually illustrated in Figures 1 and 2 in their paper. In the fluid phase, amplification is maintained primarily by C3b_2_-IgG complexes. These complexes bind properdin biva-lently and have a half-life longer than free C3b since both C3b molecules are partially protected from inactivation by fH and fI, promoting AP amplification. On cell surfaces nascent C3b binds covalently with the thioester bond to surface components forming clusters recruiting fB, fD and properdin resulting in additional convertase activity and amplification.

A common feature of the experimental models discussed above is the central importance of AP amplification. In addition, clinical evidence underlines the prominent role of complement in the pathogenesis of numerous inflammatory diseases. The list of such conditions is rapidly growing, including immune complex diseases such as rheumatoid arthritis and systemic lupus erythe-matosus, I/R injury locally manifested as infarctions or systemi-cally as a post-ischaemic inflammatory syndrome, systemic inflammatory response syndrome and acute respiratory distress syndrome, renal diseases, inflammatory and degenerative diseases in the nervous system, transplant rejection and inflammatory complications after cardiopulmonary bypass and haemodialysis. In principle, when inflammation is involved in the pathogenesis, complement has to be considered as a possible mediator in the disease process and, thus, a possible candidate for therapeutic intervention [60].

## Properdin

### Control protein in the alternative pathway amplification loop

In the long-standing model, AP is slowly activated spontaneously by hydrolysis of C3 with exposure of the internal thioester bond, and C3b is subsequently attached covalently to nearby targets. Bound C3b then binds to fB, and the resulting C3bB complex is cleaved by fD. The Ba fragment is released into the fluid phase. Bb remains as part of the C3bBb complex, the AP C3 convertase, which splits more C3 resulting in self-amplification and generation of the C5 convertase C3bBb3b. The C3Bb complex is relatively unstable with a half-life of 90 sec. under physiological conditions [61, 62]. Properdin associates with C3bBb forming a more stable C3bBbProperdin (C3bBbP) complex [63, 64] with longer lasting enzyme activity essential for effective AP amplification. Regulation of complement generally involves a number of inhibitory proteins protecting the host against tissue damage. Interestingly, properdin is the only known regulator of complement that enhances activation.

### Recognition molecule in the alternative pathway

Revision of our concepts concerning properdin as recognition molecule in AP started with experiments showing that purified properdin bound to a biosensor surface could serve as a platform for in situ assembly of AP C3 convertases [65]. In a follow-up paper, the authors stated that ‘The properdin-directed model is consistent with a proposal made by Pillemer and his colleagues >50 years ago’[27]. Pillemer and his collaborators first described properdin reacting with zymosan, a carbohydrate complex derived from yeast cell walls [66] and ‘provided evidence that properdin:target complexes directed antibody-independent complement activation’[27]. Reaction between properdin and various bacterial polysaccharides was also reported by Pillemer *et al.*[67]. The findings were difficult to reproduce, criticized, and with Pillemer's death [68–70] properdin was largely dismissed by the scientific community. The properdin system was reborn as the AP more than 20 years later [27, 69], and the current two model systems of properdin activity [27] form a valuable basis for further development in this area.

Development of properdin knockout mice [28, 29] provided additional, independent evidence indicating that properdin acts as recognition molecule, emphasizing AP as a recognition pathway [71]. LPS of *Salmonella typhosa* coated on ELISA plates was used to measure complement activation in Mg/EGTA buffer, a standard assay of AP activation. Normal wild-type mouse serum showed substantial activity, whereas properdin −/− serum showed no activity being fully restored by purified properdin.

Further studies are needed to confirm properdin as recognition molecule in AP and to delineate its fine specificity in this reaction.

The dual function of properdin is further illustrated in Figure 1.

## Influence of alternative pathway in deficiency states

### Factor H deficiency and variants

Factor H is a potent soluble inhibitor of AP by serving as cofactor for fI in conversion of active C3b to inactive iC3b. Factor H is required for keeping the physiologic, spontaneous hydrolysis of the C3 molecule under control [72]. Thus, in the absence of fH, AP will be highly activated without any particular activation substance present and activated complement will then be deposited particularly on the glomerulus basement membrane due to the fenestrated endothelial lining. Pigs genetically deficient in fH and fH −/− mice spontaneously develop membranoproliferative glomerulonephritis type II [73, 74].

It has become evident that not only fH deficiency, but also genetic variants and polymorphisms predispose to several other serious diseases like atypical haemolytic uremic syndrome and age-related macular degeneration [75–79]. A role for fH in surface protection of endothelial cells has been proposed as a pathophysiologic mechanism which may explain the predisposition of diseases related to genetic variations in the protein [80]. Furthermore, genetic variants of other complement regulators controlling the AP activation (MCP, fI and fB, the latter by ‘gain of function’) display similar disease predispositions [81, 82]. Based on the role of AP in a number of serious diseases [83], therapeutic strategies for interference with AP have been proposed [84].

### C2 bypass in CP and LP

The complement system is a typical cascade system with a strict order of activation and amplification at specific sites. In genetic deficiencies of individual components, bypass mechanisms have been identified, like ‘C2 bypass’ in C2 deficiency.

Basic observations on CP C2 deficiency were made in guinea pigs [85]. High concentrations of anti-Forssman antibody induced efficient lysis of sheep erythrocytes *in vitro* in C2 deficient serum. Inhibition experiments showed that a fully functional AP was required for haemolysis. A Forssman shock model was used to test the relevance of C2 bypass *in vivo*: intravenous injection of anti-Forssman antibody in normal guinea pigs resulted in rapid death from pulmonary shock whereas C2 deficient guinea pigs died in a delayed fashion or had a sub-lethal reaction.

LP C2 bypass in human beings has been demonstrated independently in two different model systems:

- In C2 deficient serum, specific LP activation by mannan on the solid phase in ELISA would be expected to stop at the C4 stage, while direct activation of AP might occur at high man-nan concentrations. We observed a markedly increased deposition of C3b on the solid phase at 10 ug mannan/ well compared with 0.5 ug/well. This deposition was not inhibited by anti-C1q clone 85 mAb, but completely by anti-MBL mAb 3F8 demonstrating LP C2 bypass [15]. Similar findings have later been obtained concerning deposition of TCC on the solid phase. This LP C2 bypass deposition depends on intact AP function and is completely inhibited by anti-fD (Harboe and Mollnes, unpublished observations).
- LP C2 bypass was also demonstrated by quantification of C3 deposition onto wells coated with *Salmonella* O antigen-specific oligosaccharides after incubation with normal human serum compared with C2-deficient serum. Deposition in C2-deficient serum was completely dependent on MBL and intact AP function [86].

### Properdin deficiency

The properdin gene is located on the short arm of the X chromosome, at Xp11.3-p.11.23 and is composed of 10 exons. Properdin deficiency was first described in a Swedish family in association with fulminant meningococcal disease [87]. In type I deficiency with complete absence of circulating properdin inherited as an X-linked recessive disorder [35–37], distinct mutations have been demonstrated in exon 4, 5, 6, 8 and 10 of the properdin gene.

Essential information on mechanisms of defence in meningo-coccal infection has been provided by studies of various forms of complement deficiency [88–90]. Deficiency of one of the terminal complement components leads to inadequate TCC formation and increased risk of meningococcal infection, estimated up to 6000 fold [88, 90]. Interestingly, most of these infections are caused by relatively rare serogroups and run a mild course [91]. In deficiencies of earlier components, C3b-mediated clearance of bacteria is missing and a more severe course may be expected, as often observed in properdin deficiency [87, 92]. Experiments to identify the specific targets recognized by properdin in the ‘direct model’ have begun suggesting that properdin recognizes a common bacterial surface component that is readily exposed in *Neisseria* but masked by the O-antigen in wild-type smooth enteric bacteria [27], fitting the clinical finding of frequent serious meningococcal infection in properdin deficiency.

After sub-lethal cecal ligation and puncture (CLP) as *in vivo* model for acute poly-microbial septic peritonitis, properdin deficient −/− mice showed distinctly decreased survival; in the first 7 days of the observation period, 14 of 16 wild-type mice survived, whereas only 6 of 16 properdin deficient mice survived [29].

An interesting observation regarding the role of properdin was recently recognized in two different methods for detection of AP deficiency [93]. In the traditional haemolytic assay based on human serum added to rabbit erythrocytes, properdin deficiencies were frequently missed, probably since the assay was based on the ‘self-non-self’ discrimination and triggering of the amplification loop. In contrast, a solid phase ELISA based on LPS coating of the wells [94] efficiently recognized properdin deficiencies, most likely due to the pattern recognition mechanism of AP through properdin.

## Complement in sepsis

Sepsis and accompanying systemic inflammatory response syndrome (SIRS) represent a spectrum of clinical symptoms and a complex pathophysiology. In the United States, sepsis affects about 700,000 people and accounts for about 210,000 deaths per year. The incidence of sepsis is rising, at a rate of approximately 1.5% per year despite progress in developing antibiotics, technical developments in intensive care units and other supportive care therapies. The costs of sepsis in the United States are estimated to be approximately $16.7 billion per year, a major burden to the health care system. Sepsis is a major health problem with high morbidity and a mortality in the range of 30–70%[95–97]. Sepsis is the commonest cause of neonatal mortality being responsible for 30–50% of the total neonatal death in developing countries each year [98, 99]. There are more than 200 putative mediators of sepsis and there have been more than 70 well-designed clinical trials to test the effect of manipulation of a number of these mediators. The results in large have been disappointing reflecting the complexity of the pathology [100, 101].

The complement system is activated during sepsis, leading to a systemic release of activation products with undesired effects on the host. After meningococcal infection, the clinical course shows marked variation. Meningitis may run a benign course with moderate outgrowth of meningococci in the bloodstream, while fulminant meningococcal septic shock is characterized by rapidly overwhelming intravascular outgrowth of meningococci, high concentrations of endotoxin and massive induction of proinflammatory mediators [90, 102].

We demonstrated a strong correlation between the degree of complement activation at hospital entry and the subsequent course in patients with systemic meningococcal disease [103]. Here, six of seven patients with TCC above a certain cut-off at admittance died during the course whereas only one of 32 of those with TCC below this value died (*P* < 0.0001). Using pathway-specific assays, we later showed that the overwhelming complement activation seen in those with fulminant, lethal septicaemia, was due to an uncontrolled AP amplification [104].

In experimental models of gram-negative sepsis, it has been clearly demonstrated, using specific complement inhibitors, that complement activation is of essential importance in the development of septic shock [105]. Blockade of C5a or the C5a receptor has markedly improved morbidity and mortality in rodent models of cecal ligation and puncture, leading to severe poly-microbial sepsis [106] C1-inhibitor has been used in patients with sepsis and other systemic inflammatory reactions associated with capillary leakage, but randomized clinical studies using complement inhibitors in sepsis have not been performed.

Lipopolysaccharides (LPS) have been implicated in the patho-genesis of gram-negative sepsis through their ability to stimulate synthesis of proinflammatory cytokines through binding to CD14 and the toll-like receptor 4 (TLR4)/MD-2 complex [107, 108]. LPS is known to activate complement, but large doses are needed compared to the trace amounts sufficient for cytokine production [109]. In fact, we also showed, using LPS-deficient and -sufficient *Neisseria meningtidis* strains, that membrane LPS plays a minor role in activating complement by the bacteria, in contrast to synthesis of cytokines [110]. Thus, other membrane constituents are of importance with respect to complement activation. Neutralization of LPS or blocking of CD14 has been effective in preventing lethal shock in animal studies, but results from clinical studies have been disappointing.

Complement and Toll-like receptors reacting with CD14 in complex with LPS binding protein are the two main upstream components of the innate immune system recognizing exogenous as well as endogenous ligands. They react partly independently in the inflammatory network, also having a set of cross-talk mechanisms: Complement activation may induce up-regulation of CD14 enhancing TLR4-induced inflammatory reactions [111], C1q modulates LPS/TLR-induced cytokine production [112] and MBL enhances TLR-2 and TLR-6 signalling from the phagosome [113].

In recent studies of an *Eschericia coli* induced inflammatory response in human whole blood, combined inhibition of complement and CD14 completely abrogated up-regulation of CD11b, phagocytosis and oxidative burst, whereas inhibition of each of the systems abrogated the response only partially or marginally [109]. We suggest that combined inhibition of complement and CD14 should be further explored as a treatment regimen to reduce the overwhelming damaging inflammatory response during sepsis [114].

## Concluding remarks

Properdin has two distinct functions. In addition to its well-known role to enhance AP activation by stabilization of the C3bBb complex, it is being established as a recognition factor directly initiating AP activation, like C1q in CP and MBL in L P.

AP amplification plays a decisive role for the final effect of initial activation of CP and LP, mediated through the terminal pathway products C5a and TCC. There is a need for a revised interpretation of the findings in important experimental models and human disease with greater emphasis of AP amplification.
